# An overview of commercialization and marketization of thermoelectric generators for low-temperature waste heat recovery

**DOI:** 10.1016/j.isci.2023.107874

**Published:** 2023-09-09

**Authors:** Kuan-Ting Lee, Da-Sheng Lee, Wei-Hsin Chen, Yu-Li Lin, Ding Luo, Young-Kwon Park, Argel Bandala

**Affiliations:** 1 Department of Aeronautics and Astronautics, National Cheng Kung University, Tainan 701, Taiwan; 2 Department of Chemical and Materials Engineering, Tunghai University, Taichung 407, Taiwan; 3 Department of Energy and Refrigerating Air-conditioning Engineering, National Taipei University of Technology, Taipei 106, Taiwan; 4 Research Center for Smart Sustainable Circular Economy, Tunghai University, Taichung 407, Taiwan; 5 Department of Mechanical Engineering, National Chin-Yi University of Technology, Taichung 411, Taiwan; 6 ApexGreen Technology Co., Ltd., Tainan 745, Taiwan; 7 Key Laboratory for Thermal Science and Power Engineering of Ministry of Education, Department of Engineering Mechanics, Tsinghua University, Beijing 100084, China; 8 School of Environmental Engineering, University of Seoul, Seoul 02504, Republic of Korea; 9 Department of Electronics and Communications Engineering, De La Salle University, Manila 0922, The Philippines

**Keywords:** Physics, Materials science, Economics

## Abstract

According to statistics, low-temperature waste heat below 300°C accounts for more than 89% of industrial waste heat. If the waste heat is not recycled, a large amount of low-temperature waste heat will be released into the atmosphere, thereby exacerbating global warming and posing a significant threat to human survival. Although the power generation efficiency of solid-state thermoelectric generation technology is lower than the organic Rankine cycle, it only requires a smaller construction area, which increases its market acceptance, applicability, and penetration. Especially in the pursuit of net-zero emissions by global companies, the importance of low-temperature waste heat recovery and power generation is even more prominent. The current thermoelectric conversion efficiency of commercial thermoelectric chips is about 5%. Power generation cost, thermoelectric conversion efficiency, and energy use efficiency are highly correlated with the commercialization of solid-state thermoelectric technology. This research shares five practical waste heat power generation cases commercialized by recycling three heat sources. It also points out the three significant challenges facing the commercialization of power generation from low-temperature waste heat recovery. This study analyzes 2,365 TEG patents submitted by 28 companies worldwide to determine the basic technology for realizing waste heat recovery through TEG and explore the potential commercialization of related waste heat recovery products. The future challenge for the large-scale commercialization of solid-state thermoelectric technology is not technological development but financial incentives related to changes in international energy prices and subsidies that promote zero carbon emissions.

## Graphical abstract

**Figure:**
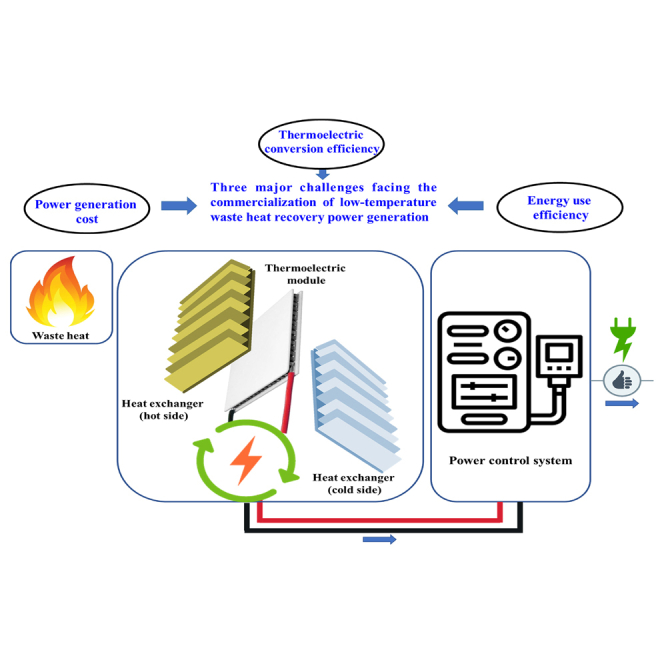

## Introduction

**Figure 1:**
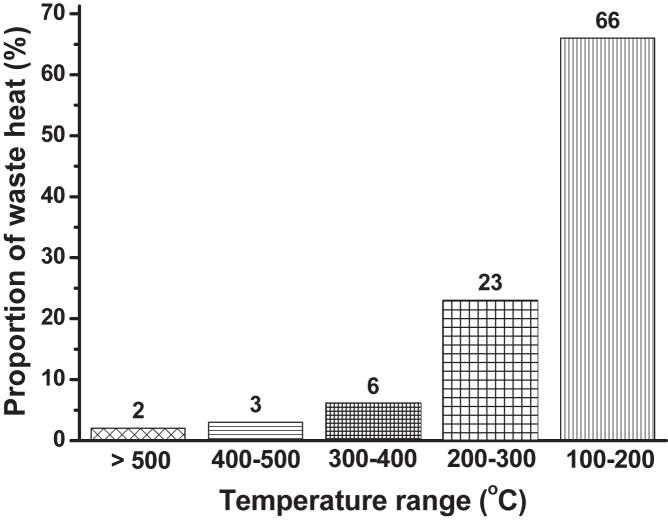
Waste heat by temperature range (License number: 5386870154504)^3^

Heat energy is the most commonly used form of energy in industry, accounting for 90% of total energy usage.^1^ Waste heat will exist and be liberated because heat energy is the final residual form. Manufacturing is the largest energy-consuming industry in various countries and produces large amounts of waste heat through steam boilers, incinerators, and heating furnaces.^2^ Figure 1 presents the distribution of industrial waste heat by temperature range. Waste heat can be divided into three categories: high temperature (above 500°C), medium temperature (300°C–500°C), and low temperature (below 300°C).^4^ The higher the temperature, the higher the quality of waste heat. Notably, most high-grade waste heat >300°C can be recovered.^5^ EU states that low-temperature waste heat makes up 89% of industrial waste heat, with over 66% below 200°C.^6^^,^^7^ This means that if waste heat is not recycled, a large amount of low-temperature waste heat will be released into the atmosphere, thus exacerbating global warming, producing extreme climatic events, and causing significant crises that threaten the survival of humankind.

Waste heat refers to the waste heat that has been recycled once or cannot be recycled.^3^ Although waste heat cannot be directly converted to usable energy, it is a part of energy reuse processes in the manufacturing industry. Increasing market penetration, optimizing heat conversion efficiency, and establishing a supply chain system are priorities for countries seeking to develop waste heat recovery and reuse capabilities. PetroChina Liaoyang Petrochemical Company developed a low-temperature waste heat recovery system (WHRS) in 2020 that can generate approximately 2,800 kWh per hour. This system has the largest waste heat recovery scale, the highest single-unit power generation capacity, and the highest comprehensive energy utilization rate among similar systems in China. This system can save approximately 120,000 tons of raw coal, 205,000 tons of carbon dioxide, and CNY 100,000,000 each year.^8^

Two technologies can be used for low-temperature waste heat recovery and power generation. The first is the organic Rankine cycle (ORC), driven by a working fluid with a low boiling point, such as a refrigerant.^9^^,^^10^ Although the ORC offers high power generation efficiency, it requires a large construction area. The second is a solid-state thermoelectric technology that uses specific materials to directly convert thermal energy into electrical energy. Although its power generation efficiency is lower than that of ORC, it requires a small construction area. Power generation requiring more than 50 kW will generally require the ORC system. Otherwise, solid-state thermoelectric technology would be recommended. Solid-state thermoelectric tech can recycle multiple heat sources and overcome space limitations in industry. Its high penetration rate makes it a valuable solution for using large-scale equipment.

The widespread industrial adoption of thermoelectric power generation reveals its increasingly crucial role as a green energy technology.^11^ The current thermoelectric conversion efficiency of commercial thermoelectric chips is about 5%. Among commercially used thermoelectric materials, the figure of merit (ZT) of adopted Bi_2_Te_3_ is approaching 1.2.^12^ In contrast, PbTe, GeTe, and SiGe are the mainstream development material in medium- and high-temperature ranges. Recently, some applications, such as flexible and wearable thermoelectric devices, have been reported.^13^ Due to their solid-state conversion capabilities between heat and electricity, zero emission, and high flexibility, flexible thermoelectric devices have exhibited great application possibilities for portable power generation and localized refrigeration.^14^ Advanced countries such as the United States and Japan have launched large-scale thermoelectric research and development projects supported through government funding. U.S. Department of Energy collaborates with enterprises to develop materials for high-temperature exhaust waste heat recovery from automobile engines.^15^^,^^16^ Japan’s New Energy and Industry Technology Development Organization endorses thermoelectric technology for automobile engines and waste heat recovery.^17^^,^^18^ South Korea established a research center for advanced hybrid electric vehicle energy recovery systems;^19^ China invested CNY 30 million to develop thermoelectric materials.^20^

The literature survey suggests that many review papers concerning thermoelectric (TE) generator (TEG) and generation have been published, with emphasis on the academic research of TE material preparation, TEG design, and TE system operation.^5^^,^^21^^,^^22^^,^^23^ However, no review paper concerning TEG commercialization and marketization has been published yet, and this topic is crucial in the industrial development and applications of TEG. For this reason, the present study aims to provide a comprehensive review of TEG commercialization and marketization. The obtained results can provide helpful insights into TEG research and applications.

## Thermoelectric technologies

**Figure 2:**
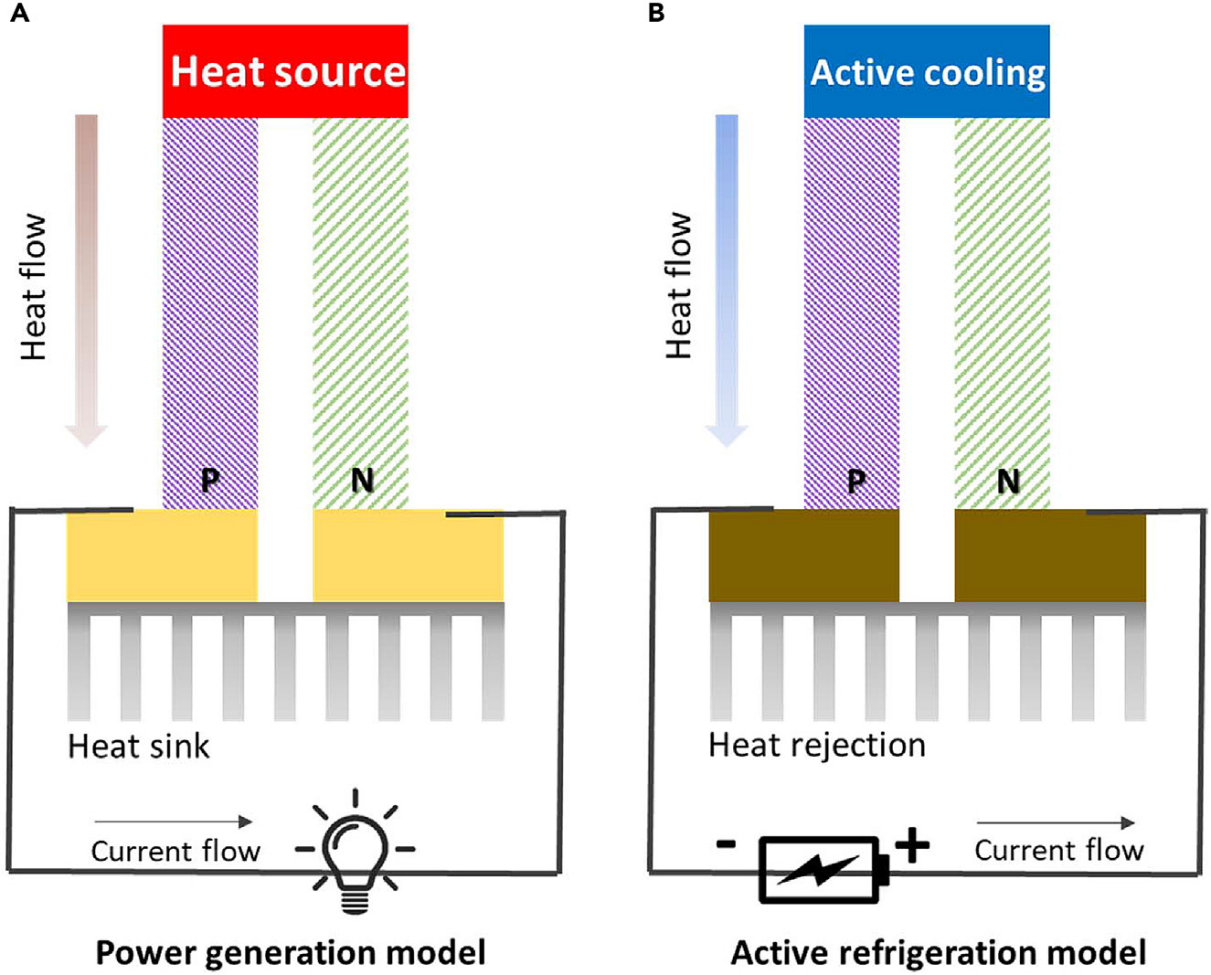
Thermoelectric generator and cooler Thermoelectric module for (A) power generation and (B) active refrigeration.

Solid-state thermoelectric technology, known as thermoelectric technology, is an energy conversion technology without motion behavior. According to statistics on thermoelectric applications, more than 6,900 articles have been published in the past five years. It is found that since 2019, the number of articles has exceeded 1,000 each year, and the publication rate is increasing at an annual rate of more than 13%. Thermoelectric technology involves two modes of operation: power generation and refrigeration, as shown in Figure 2. Thermoelectric generation technology is a static power generation technology similar to solar photovoltaic technology. When the external environment causes a temperature difference in a thermoelectric material, and heat energy passes through the material, the heat energy creates a potential difference because of the material’s characteristics, and the material can operate under an external load to generate electricity.^24^ However, if electric energy is input into the thermoelectric material, the material can extract and dissipate heat in a similar manner as a heat pump; this is called the thermoelectric cooling or heat pump phenomenon.

Solid-state thermoelectric technology is based on a thermoelectric module consisting of several thermoelectric materials and can be expanded to any scale through series and parallel connections. It has high device flexibility and can be used in heat sources of various scales, especially in cases of large amounts and multiple types of industrial waste heat. Because the module’s parts do not move, as long as the temperature remains within a certain range, the thermoelectric material is durable, and its maintenance cost is low. The flue gas emission temperature is usually below 300°C in the industrial boiler market.^25^ For higher-temperature waste heat, cooling methods can reduce the heat to the temperature range recycled by low-temperature thermoelectric materials. However, several mature and effective high-temperature waste heat recovery technologies, such as cogeneration technology, have been developed.^26^ Because heat energy is not naturally converted directly into electrical energy, this process cannot occur without purposeful manipulation and is more complicated than burning fuel to release energy. Solid-state thermoelectric power generation technology has a low heat transition efficiency and high cost. Therefore, it is mainly used in niche applications, such as the power supply for remote sensors, etc.^27^^,^^28^ However, if the scale of the system were expanded, technical and business model analysis suggests its cost would be comparable to that of general electricity.

### Thermoelectric materials

**Figure 3:**
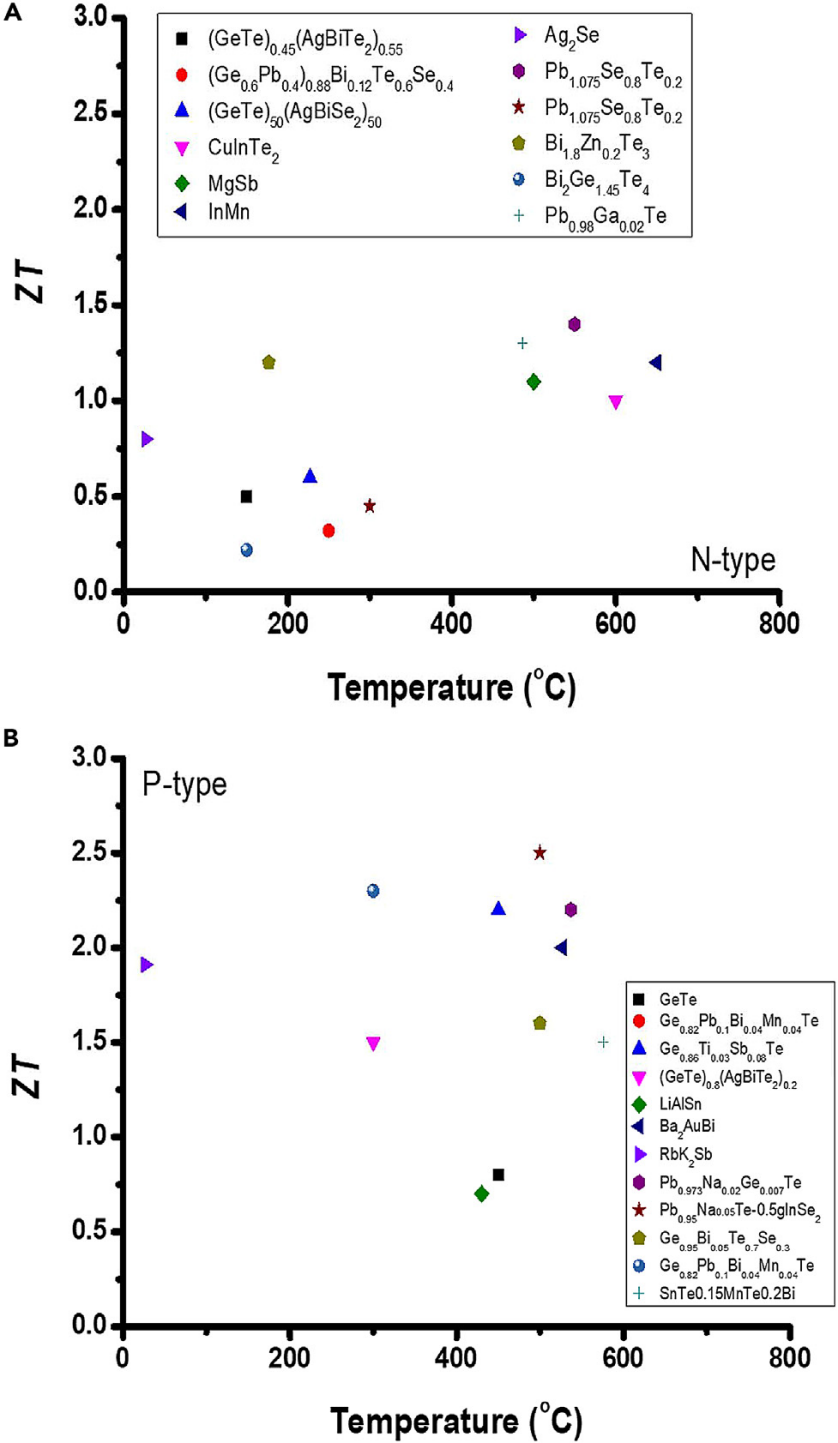
Materials’ temperature-dependence ZT values Temperature dependence of *ZT* for (A) N-type thermoelectric materials of (GeTe)_0.45_(AgBiTe_2_)_0.55_,^38^ (Ge_0.6_Pb_0.4_)_0.88_Bi_0.12_Te_0.6_Se_0.4_,^38^ (GeTe)_50_(AgBiSe_2_)_50_,^38^ CuInTe_2_,^39^ MgSb,^40^ InMn,^41^ Ag_2_Se,^42^ Pb_1.075_Se_0.8_Te_0.2_,^43^ Bi_1.8_Zn_0.2_Te_3_,^44^ Bi_2_Ge_1.45_Te_4_,^45^ and Pb_0.98_Ga_0.02_Te^46^ and (B) P-type thermoelectric materials of GeTe,^38^ Ge_0.82_Pb_0.1_Bi_0.04_Mn_0.04_Te,^38^ Ge_0.86_Ti_0.03_Sb_0.08_Te,^38^ (GeTe)_0.8_(AgBiTe_2_)_0.2_,^38^ LiAlSn,^47^ Ba_2_AuBi,^48^ RbK_2_Sb,^49^ Pb_0.973_Na_0.02_Ge_0.007_Te,^46^ Pb_0.95_Na_0.05_Te-0.5% AgInSe_2_,^50^ Ge_0.95_Bi_0.05_Te_0.7_Se_0.3_,^51^ Ge_0.82_Pb_0.1_Bi_0.04_Mn_0.04_Te,^52^ and SnTe0.15%MnTe0.2%Bi.^53^

Thermoelectric efficiency can be increased by adjusting materials, modules, and systems. Several significant factors, such as electrical conductivity, thermal conductivity, and the Seebeck coefficient, influence the efficiency of TE modules. Improving the ZT value by manipulating a single factor is a challenging task. Modulation doping and band engineering techniques are usually employed to increase electrical conductivity. On the other hand, improving the Seebeck coefficient can be achieved through the energy-filtering effect, resonant levels, ionization scattering, and band convergence. Furthermore, the thermal conductivity of thermoelectric materials can be successfully reduced by enhancing phonon scattering.^29^ Materials with high electrical conductivity and low thermal conductivity are crucial to the efficiency of thermoelectric modules. Solid substances that conduct electricity usually conduct heat and vice versa. For example, metals conduct electricity and heat, whereas plastics do not; this is known as “one-way behavior.” However, effective thermoelectric materials must have adequate electrical conductivity and low thermal conductivity; this is called “reverse behavior.” The *ZT* can determine the efficiency of thermoelectric material-based devices.^30^^,^^31^^,^^32^ The dimensionless figure of merit of a thermoelectric material depends on the relationship between its electric conductivity, thermal conductivity, and Seebeck coefficient and is calculated as follows:

(Equation 1) $$ZT=\frac{\sigma \times S^{2}}{K}\times T$$

where *σ*, *S*, *K*, and *T* represent the electric conductivity, Seebeck coefficient, thermal conductivity, and absolute temperature, respectively. *ZT* is a function of temperature (*T*). During charge carrier excitation, electron-hole pairs produced under certain temperatures in a thermoelectric material result in a bipolar effect that reduces the Seebeck coefficient and increases thermal conductivity. Therefore, thermoelectric materials usually cannot be used under a wide temperature range. Bi_2_Te_3_ is a traditional thermoelectric material. Due to the limitations of its material properties, the operating temperature is difficult to exceed 250°C. Otherwise, it will lead to a decrease in ZT value. Recently, a series of thermoelectric materials with an operating temperature from near-room temperature to 300°C (RT-300°C) have been reported, such as MgAgSb^33^ and Mg_3_Sb_2_.^34^ Their figure of merit of *ZT*_max_ is about 1.3.^33^ A thermoelectric material for a specific temperature must have high electrical conductivity, a high Seebeck coefficient, and low thermal conductivity, which produce high *ZT* values. Commercial thermoelectric materials have a *ZT* value of approximately 1,^35^ whereas thermoelectric materials with a *ZT* value of approximately 1.5 are in the laboratory development stage.^36^ Thermoelectric materials can be divided into three categories on the basis of operating temperature: low temperature (25°C–300°C), medium temperature (300°C–600°C), and high temperature (above 600°C).^37^ Figure 3 shows the ZT of various thermoelectric alloy materials in recent years at various temperatures, including BiTe, PbTe, GeTe, Ag_2_Se, MgSb, RbSb, LiAlSn, InMn, etc.^54^ Summarizing the ZT of these thermoelectric materials in recent years, BiTe-based thermoelectric materials have the most development potential in the low-temperature range. In contrast, PbTe, GeTe, and SiGe are the mainstream development material in medium- and high-temperature ranges.

The representative thermoelectric materials for each temperature range are low-temperature BiTe series,^55^ medium-temperature PbTe series,^56^ and high-temperature SiGe series.^57^ Bi_2_Te_3_ is the only material that has matured and been commercialized for use in thermoelectric modules. Because of its ability to convert waste heat energy into beneficial forms of electrical energy, it is a highly effective thermoelectric material. The physical behavior of Bi is similar to that of metal. When Bi is alloyed with Te to become Bi_2_Te_3_, it behaves similarly to an effective semiconductor thermoelectric material. The *ZT* of commercialized Bi_2_Te_3_ is 1–1.2.^12^ To optimize the thermoelectric conversion efficiency of Bi_2_Te_3_ and improve the quality of thermoelectric materials, numerous studies have shifted development toward the nanometerization of thermoelectric materials, developing nanorods,^58^ nanowires,^59^ nanoplates,^55^ nanotubes,^60^ nanoflowers,^61^ and nanosheets.^62^ According to experimental results in the literature, although the nanometerization^63^ of thermoelectric materials, alloying,^64^ entropy engineering,^65^ and band engineering^66^ can help increase thermoelectric conversion efficiency, the consistency and reproducibility of its performance remain to be determined. Given current market demand, the commercially available low-temperature thermoelectric materials are sufficient to manage most industrial waste heat.

### Thermoelectric modules

**Figure 4:**
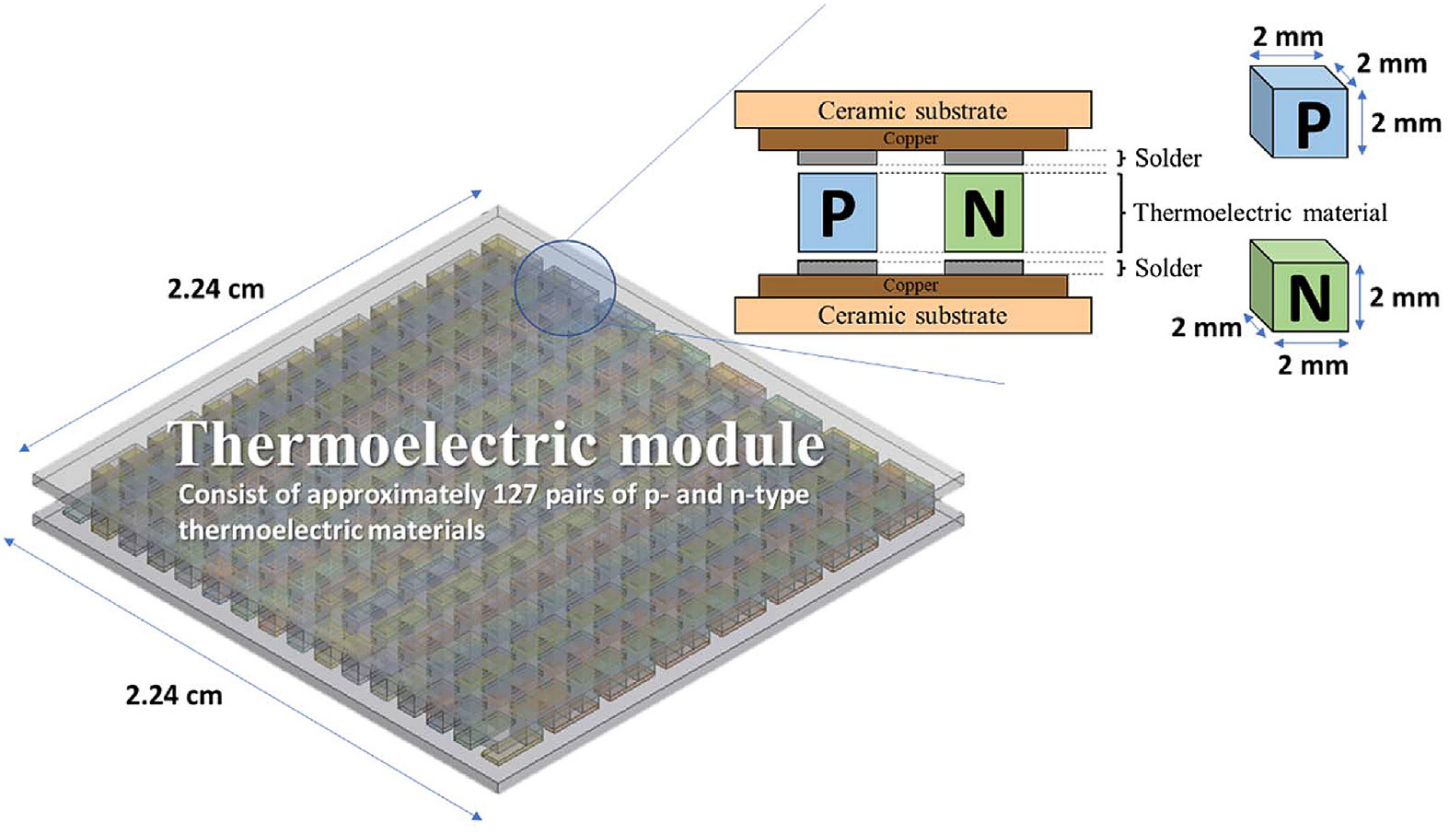
Illustration of the thermoelectric module

A thermoelectric module consists of thermoelectric materials. The area of a typical commercially available thermoelectric module is about 5 cm^2^, and the thickness would be approximately 5 mm, as shown in Figure 4. The internal thermoelectric materials’ length, width, and height would be 1–3 mm, and a module could contain tens to hundreds of thermoelectric materials. Commercially available thermoelectric modules consist of approximately 127 pairs of p- and n-type thermoelectric materials.^67^ A single thermoelectric material (cuboid) is around 2 mm in length, 2 mm in width, and 2 mm in height.^68^ Thermoelectric modules consist of five units: (1) a thermoelectric material generating electron-hole pairs, (2) a ceramic substrate used as the main support structure to receive cold and heat sources and prevent power loss, (3) electrode-connecting circuits, (4) a solder for soldering the thermoelectric material to the electrode, and (5) a diffusion barrier to prevent intermetal diffusion between materials and solders under high-temperature conditions. Modules also contain two wires that output the generated electricity. The solder in thermoelectric modules is usually tin alloy and has a melting point of approximately 250°C.^69^ Therefore, the upper limit for the operating temperature of thermoelectric modules is generally 200°C–230°C to prevent damage to the component structure due to overheating the solder. Thermoelectric modules are easy to use. Heat energy can be transferred from the high-temperature side to the low-temperature side of the module by connecting both sides to a heat exchanger; thus, the module generates a temperature difference and electricity. In terms of *ZT* values, the performance of commercially available thermoelectric modules is approximately 80% that of their thermoelectric materials, meaning that when a thermoelectric material with a *ZT* of 1 is used in a thermoelectric module, the resulting *ZT* value of thermoelectric module would be approximately 0.8. Numerous studies on thermoelectric modules have explored the size and shape of the thermoelectric materials used in thermoelectric modules.^70^^,^^71^^,^^72^

### Thermoelectric systems

Thermoelectric systems are essential to the commercial operation of thermoelectric modules. Thermoelectric power generation systems consist of three parts: a heat exchanger (hot side), a thermoelectric module, and a heat sink (cold side). Before the heat source enters the thermoelectric module, the heat exchanger evenly conducts waste heat to the surface of the module. The heat from the heat source is absorbed through two processes. For medium- and low-temperature heat sources, the heat exchanger directly contacts the surface of the heat source and transfers the heat to the thermoelectric module. For high-temperature heat sources (such as the heat source generated by the steel and papermaking industry), heat-absorbing materials are used to absorb thermal radiation energy, and the heat is indirectly transferred to the thermoelectric module to generate electricity. Thermal radiation can produce electromagnetic waves of thermal radiation wavelengths that differ depending on temperature. Therefore, heat radiation wavelength is crucial for the absorbing material to transfer heat energy to the thermoelectric module. The heat sink on the cold side plays a key role in the thermoelectric module by dissipating heat.^73^^,^^74^^,^^75^ If the heat dissipation efficiency of the radiator is low, the thermoelectric conversion efficiency will be strongly affected because of the small temperature difference between the cold and thermal measurements, which can even damage the thermoelectric module. In addition, because thermal energy is an unstable energy source, the electric energy generated by the thermoelectric system necessitates a power control system for stabilizing the output, which is an indispensable component of the thermoelectric system. Because thermoelectric systems involve numerous factors affecting conversion optimization, few studies and review articles have investigated this topic.

## Application cases

In thermoelectric generation, the device is composed of a cold side for heat dissipation and a hot side for heat supply. The heat exchanger in the device serves as an interface through outward heat flux, and the characteristics and operation modes of the cold and hot sides affect the effectiveness of thermoelectric generation considerably. Several methods of energy exchange between the cold and hot sides are used. The methods for the cold side are (a) air cooling,^76^ which is most commonly achieved through aluminum heat sinks for heat dissipation and (b) water cooling,^77^ which is frequently applied in water recirculation for factory cooling purposes. Methods for the hot side are (a) direct surface contact,^78^ which involves adhering a heat source to the surface of a heat pipe, (b) the use of self-heating fluids,^79^ such as high-temperature gases generated from burning or steam, and (c) heat radiation,^80^ which uses the heat generated from processes such as steel manufacturing.

The heat source determines the feasibility of thermoelectric generation. Heat dissipation is considered a flexible factor, meaning that various heat dissipation methods can be used for thermoelectric generation. For example, when recirculating cold water is unavailable, air cooling and other water-cooling methods can be applied instead. Air cooling is considerably less effective than water cooling in heat dissipation. Water-cooling systems are suitable for stationary thermoelectric generation applications because the energy cost of installing extra water-cooling systems is lower than that of using air-cooling systems alone. The price difference is more significant for low-temperature TEG modules.

Few cases of applying thermoelectric generation technology to low-temperature electrical generation from industrial waste heat have been documented, and relevant applications at the kilowatt scale or above are particularly rare. Thermoelectric generation entails potential commercial opportunities, and collaborative projects between industries and research institutions in Taiwan have produced several types of industrial thermoelectric generation systems that use various heat sources. The following section introduces several examples of applications in Taiwan, organized by the heat source. Most examples are from factories or locations with a supply of cooling water. Therefore, cool water was used to increase the effectiveness of electrical generation.

### Heat source: steam and hot water

#### Case 1: residual heat of recycled water from steam boilers

Saturated steam of ≥170°C generated from steam boilers is subjected to the manufacturing process before its temperature is reduced to <160°C. In the original procedure, the recirculating steam is transferred to the oxygen scavenger, and the steam pressure is increased by using a pump before it is transferred back to the boiler for heating. However, the oxygen scavenger is not sealed off from the ambient environment, causing the temperature of the recirculating steam to decrease to <100°C. This leads to a considerable thermal energy loss compared to saturated steam.

**Figure 5:**
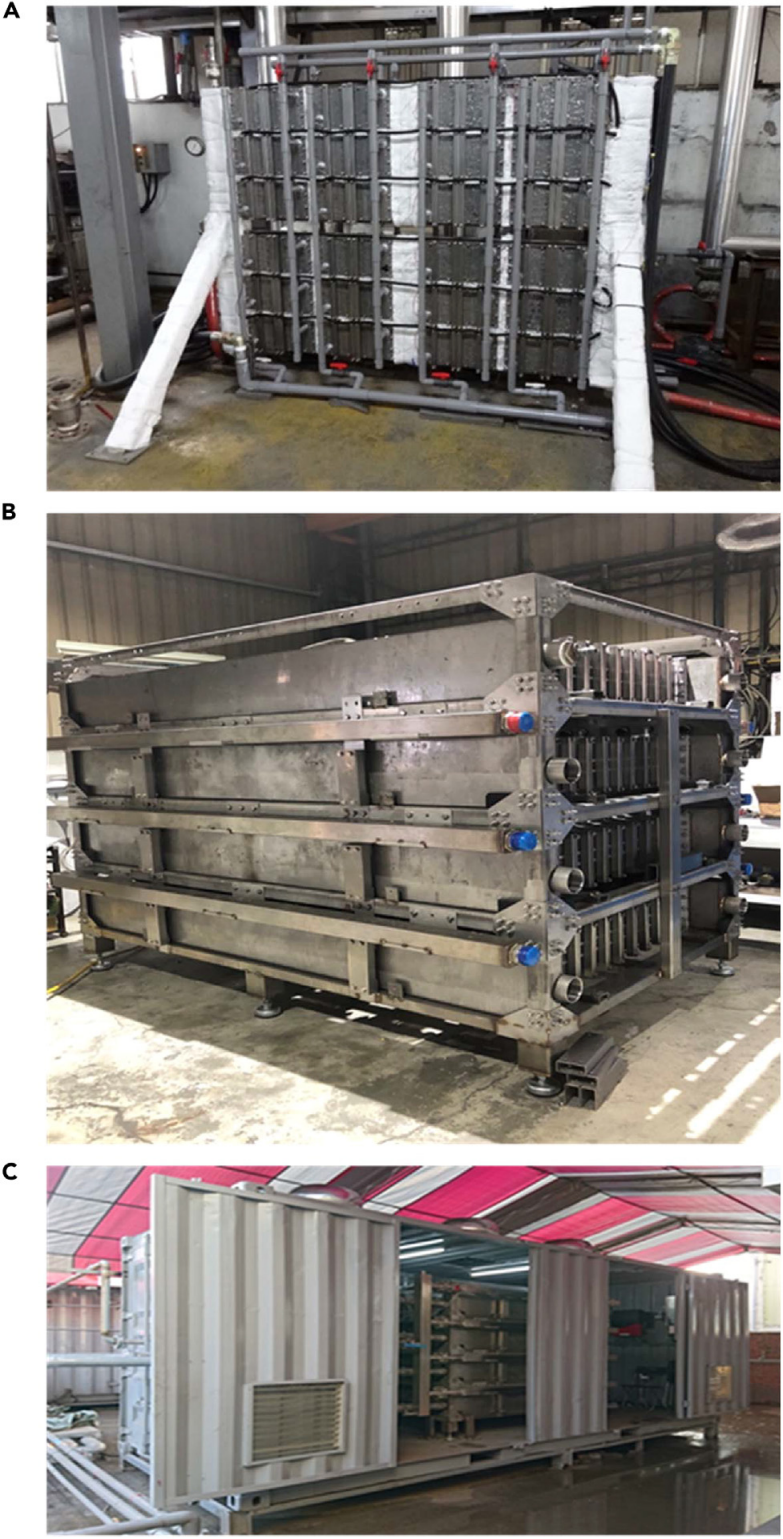
Practical thermoelectric generation systems Thermoelectric generation systems of (A) 5 kW and (B) 10 kW and (C) loading the 10-kW system into a container.

Figure 5 presents the exterior of the thermoelectric generation system. It consists of 48 small power generator units, and each power generation unit contains 32 pieces of BiTe power generation modules; that is to say, the thermoelectric power generation system uses 1,536 thermoelectric modules. The designed maximum generation is 10 kW (at 170°C of hot side temperature). The system is thin, measuring approximately 2 m (length) × 1.8 m (height) × 0.25 m (width), and therefore requires little space. System testing revealed that when the mean temperatures of the hot-end and cold-end heat exchangers were 151°C and 41°C, respectively, the maximum energy generated was 5,038 W, indicating that the system can generate sufficient power. The generated power is transmitted to the factory through power inverters and directly integrated into the power grid. This is the first case of a kilowatt-level thermoelectric generation system being successfully integrated into a grid in Taiwan. The installation structure of this system occupies a small area, highlighting the advantages that the thermoelectric system can be flexibly installed and is relatively free from environmental space restrictions.

#### Case 2: low-temperature waste heat from the waste treatment industry

This case involves a waste processing plant where plasma gasification is used as an alternative to conventional incineration. Incineration is a highly polluting thermochemical treatment process.^81^^,^^82^^,^^83^ Plasma gasification can decompose solid organic waste such as plastics to generate large quantities of combustible gases (e.g., C_x_H_y_ and CO), which are then used as fuels.^84^^,^^85^^,^^86^ The gaseous fuels are combined with saturated steam from boilers to power the thermoelectric generation system of the plant.

Figure 5C presents the thermoelectric generation system, which is loaded into a container for transportation when required. The system consists of a low-temperature BiTe TEG module comprising 32 sub-genset systems. Each subsystem contains 64 pieces of thermoelectric modules; that is, the whole system uses a total of 2,048 pieces. There are four steam heat source inlets on the thermoelectric system, and each heat source inlet provides eight subsystems to use. The designed maximum energy generation is 15 kW (at 170°C of hot side temperature). The system is approximately 2.4 m (length) × 1.8 m (height) × 1.6 m (width). System testing revealed that when the mean temperature of the hot-end and cold-end heat exchangers was 149°C and 30°C, respectively, the maximum energy generation was 13,590 W. This system is Taiwan’s first >10-kW thermoelectric generation system. However, compared with case 1, case 2 has a larger power generation capacity but requires a higher installation space. Therefore, from the commercialization perspective, it should still use smaller and higher efficiency heat exchangers to stand out the advantages of thermoelectric technology.

#### Case 3: power generation using hot springs

Taiwan is located in the Pacific Ring of Fire. Because of its unique geological features, Taiwan possesses a wealth of geothermal resources, most of which have not been adequately developed. Hot springs are one such type of geothermal resource. In Taiwan, the temperature of hot springs is approximately 100°C, which is suitable for thermoelectric generation without the risk of overheating. The Taiwanese government provides electricity bill subsidies for geothermal generation, and geothermal generators do not produce noise or negatively impact tourism in the areas in which they are used. Various incentives to adopt this generation method are also offered.

**Figure 6:**
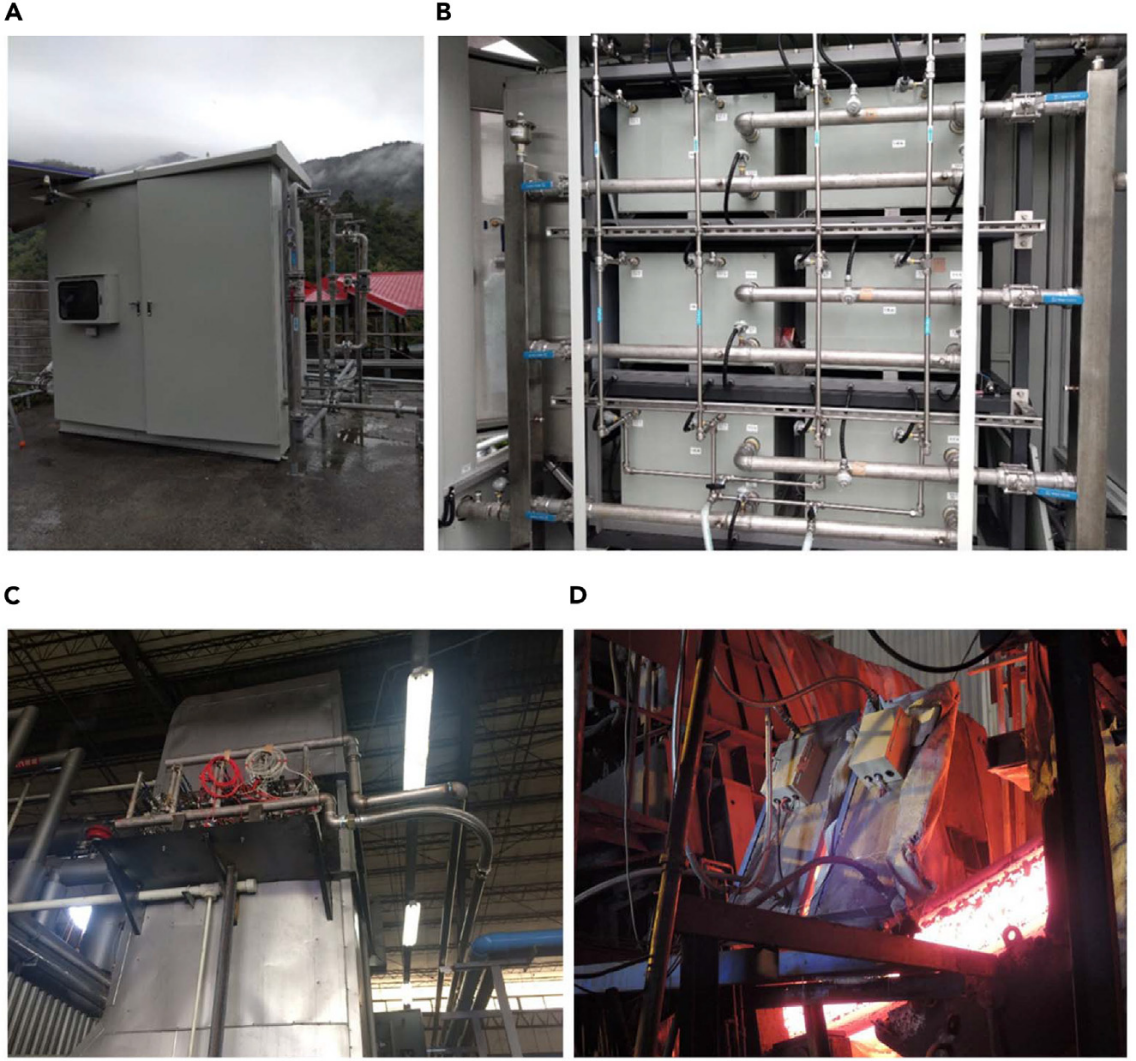
Hot spring geothermal generation system (A) Outer appearance; (B) inner appearance. Thermoelectric power generation system. (C) Heat pipe; (D) heat radiation based.

The system is in a hot spring hotel in Taitung County, Taiwan. The hotel’s hot springs can reach approximately 125°C. The temperature of the spring water from the mountains that the hotel uses to adjust the temperature of the hot springs is approximately 15°C–20°C, meaning that the mountain spring water can be directly used as the cold-side cooling water. Because the mountain spring water is mixed with the hot spring water, the unconverted residual thermal energy absorbed by the mountain spring water is not wasted. Figure 6 presents the system’s outer and inner appearance, consisting of six sub-genset systems. There are 128 pieces of BiTe thermoelectric modules in each subsystem. Regarding heat source configuration, every two subsystems are equipped with a hot spring input water pipe. Therefore, after the hot spring enters the system, it is divided into three pipelines and sent to the subsystem, and the cooling water is configured in the same way. System testing revealed that when the hot spring and cooling water temperatures were 112°C and 20°C, respectively, the power generation was 1,046 W. When the hot spring temperature was increased to 124°C, and the cooling water temperature remained at 20°C, 1,597 W of power was generated. This is Taiwan’s largest hot spring thermoelectric power generation demonstration system so far. However, since the heat source conditions of hot springs are relatively low for thermoelectricity, the power generation will be relatively low. Therefore, the thermoelectric system used for hot springs in the future should be oriented toward a more compact size. In addition, attention must also be paid to the problem of the decrease in heat exchange efficiency caused by fouling.

### Heat source: exhaust from combustion

This case focuses on recycling exhaust from steam boilers (using natural gas as the fuel). Exhaust from fossil fuel burning is the most common industrial waste heat source. The exhaust pressure of conventional combustion equipment is low. Consequently, the pressure drop caused by the heat exchanger during heat recovery must be carefully evaluated when an air extractor is not installed on the exhaust pipe outlet. This is because pressure drop in the equipment can affect the combustion quality at the front end of the exhaust equipment. In this case, smoke from a steam boiler powered by natural gas is used as the heat source. Heat pipes are used as thermal conductors to replace conventional heat sinks. This considerably increases the quantity of heat recovered from such a small space, and the resulting pressure drop is less than that caused by heat sinks with the same heat recovery capacity. Figure 6C presents the system. The surface of the rectangular exhaust pipe has a rectangular opening, and a thermoelectric generation system of the same size as the opening is placed in the pipe. The system comprises 16 heat tubes and a low-temperature BiTe TEG module comprising two sub-genset systems. At the same time, each subsystem contains 256 pieces of thermoelectric modules. The maximum energy generation is 1 kW (at 150°C of hot side temperature). System testing revealed that when the exhaust and cool water temperatures were 220°C and 30°C, respectively, the maximum power generation was 920 W. However, the system’s specialized water cooler consumes 400 W of power; thus, the system yields a net power output of 520 W.

### Heat source: heat radiation in a billet steel manufacturing facility

Waste heat is often generated from industrial heat radiation, particularly from steel, cement, and glass manufacturing processes and other thermal processing procedures. Because these manufacturing processes involve high temperatures, heat radiation is the main waste heat source. Specially treated metals or metal oxides are used to recover heat from heat radiation because such materials increase the heat absorption rate and reduce surface emissivity. Because high-temperature TEG modules are costly, the temperature of the heat recovery interface must be designed to reduce to that required by a low-temperature BiTe TEG module.

This case involves a thermoelectric generation system in a billet steel manufacturing facility (continuous casting). The surface of the billet steel can reach 1,000°C, and therefore, the specially treated surface is used as the interface to recover heat from heat radiation. A low-temperature BiTi TEG module and a water-cooling heat exchanger are installed on the back of the interface as a planar type of thermoelectric generation unit, as shown in Figure 6D. The unit is next to the billet steel channel to directly absorb the high-temperature heat radiation generated from the billet steel surface. The system comprises four such units. System testing revealed that the maximum power generation of each unit was 250–255 W; they generated similar amounts of power when exposed to the same amount of heat radiation. Accordingly, the total power generation was approximately 1 kW. However, when the high-temperature heat radiation of the steel industry is used as the heat source, it is necessary to prevent overheating the hot end of the thermoelectric module especially. In addition, the temperature fluctuation of the heat radiation source is large, which can easily cause thermal shock to the thermoelectric module and causes damage. The device design must be taken into consideration.

## Techno-economic assessment

Thermoelectric generation uses thermal energy as the energy source. Under most circumstances, the consumed thermal energy comes from waste heat with no reuse value. Therefore, there is no energy source cost when thermoelectric generation is implemented. Instead, equipment installation costs are prioritized. Problems related to the low efficiency and high costs of thermoelectric generation technology are attributable to the immature development of its core components, ranging from thermoelectric materials to power generation modules. Consequently, to date, thermoelectric generation technology is insufficient to compete with other similar technologies.

The low efficiency of thermoelectric generation prevents the heat source from being utilized efficiently and increasing the heat dissipation capacity of the cold side (e.g., increasing the volume of cooling water) to remove a large amount of unused thermal energy and maintain the temperature difference between the cold and hot sides is costly. The temperature of thermal energy emitted from the cold side is too cold to be reused in most instances (except in unique situations, for instance, in cold geographic areas, waste heat emitted from the cold end can be used to heat houses), which results in considerable energy loss and no environmental benefit. The high cost of thermoelectric generation decreases investors’ motivation to invest in the technology and prevents it from having a competitive advantage over other generation technologies. In addition to the cost of the raw materials (e.g., Bi and Te), the high thermoelectric generation cost is due to its low thermoelectric conversion efficiency. This means that the quantity of power generated per unit of raw material expended is low, resulting in an unsatisfactory price-to-performance ratio (generation cost versus volume).

A satisfactory price-to-performance ratio is key to the commercialization of thermoelectric generation; the ratio can be evaluated, with the results indicating whether to optimize the generation cost or increase the conversion efficiency. Increasing conversion efficiency is crucial to maintaining the balance between economic benefit and energy use efficiency and enhancing thermoelectric generation’s benefits. Therefore, when thermoelectric generation technology must be commercialized within a short period, decreasing the generation cost to achieve a reasonable price-to-performance ratio should be prioritized. With the existing technology, large-scale manufacturing of thermoelectric generation equipment through methods similar to those used to develop photovoltaic products is the most effective approach to reducing the generation cost.

**Figure 7:**
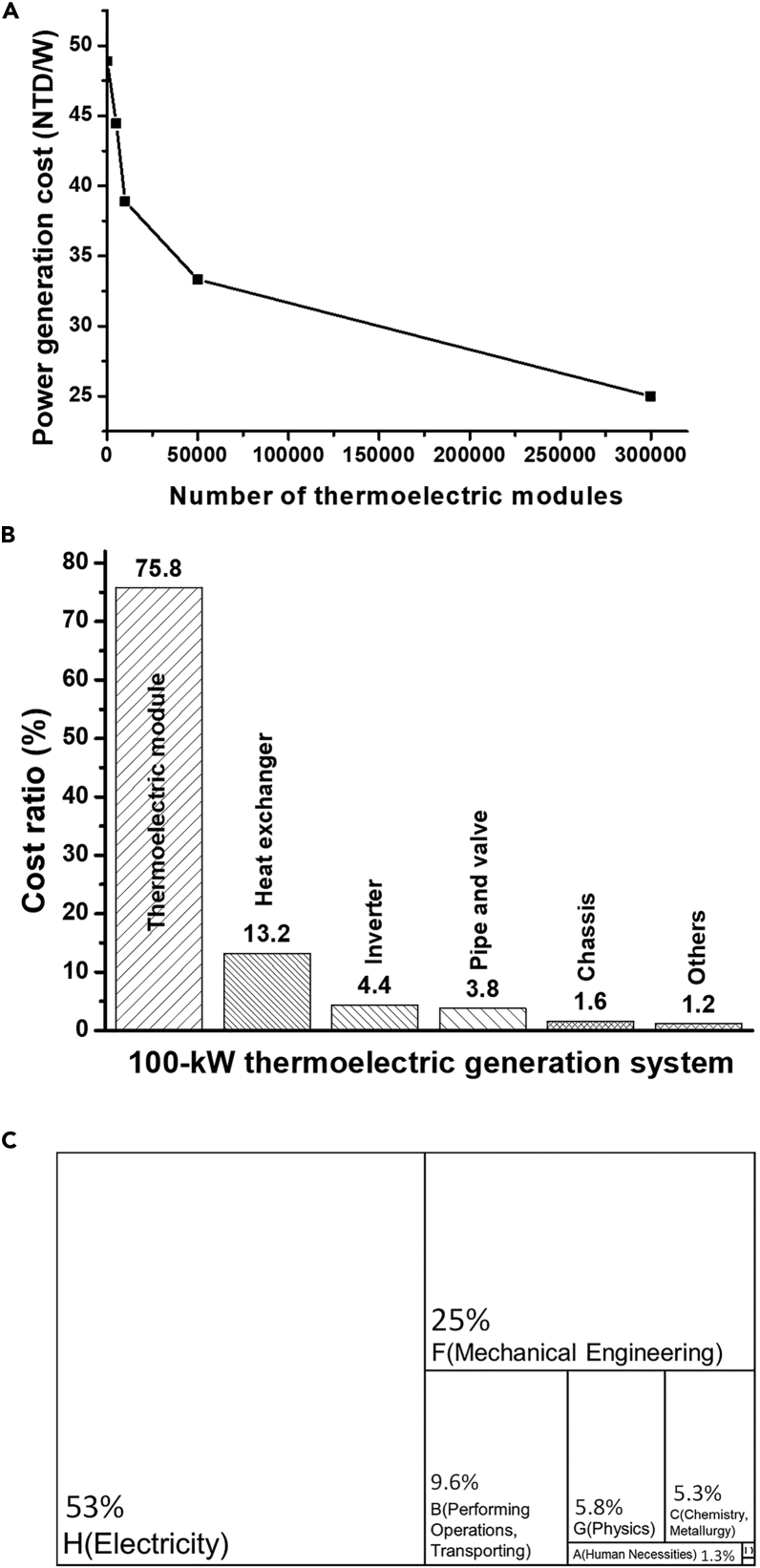
Economicanalysis and patent classification of thermoelectric systems (A) Relationship between the number of commercial low-temperature BiTe thermoelectric modules and cost per watt of power generated. (B) Cost breakdown of a 100-kW thermoelectric generation system. (C) Classification of patents related to TEG-based low-temperature WHRSs according to IPC.

A Taiwanese research institution focusing on thermoelectric generation technology collaborated with a local distributor of an international thermoelectric generation equipment supplier to analyze the selling price of TEG modules. Figure 7A presents the relationship between the generation cost per watt and the number of TEG modules under the conditions that the maximum generation of each module is 18 W and the temperature difference between the cold and hot ends is < 210°C. When 5,000 modules are installed, the cost of the module per watt of power generated is US$1.5, and when the number of modules is 10,000, 50,000, and 300,000, the cost per watt decreases to US$1.3, US$1.1, and US$0.8, respectively. This means that when 300,000 modules are installed, the cost per watt is nearly half of the cost when only 5,000 modules are installed. Compared with other power generation schemes, 300,000 is a small number of modules and can substantially reduce the total generation cost. This indicates the necessity of manufacturing TEG modules in large quantities.

Figure 7B presents the cost breakdown of a large-scale 100-kW thermoelectric generation system; the Taiwanese research institution created the cost breakdown using each part’s market price. This system requires 12,000 TEG modules. Although TEG modules are the key components in the system, they are less developed than the other components, resulting in a higher cost. Consequently, the modules account for 75% of the total cost of the thermoelectric generation system, thereby accounting for a considerably higher cost proportion than the other components.

When a thermoelectric generation system comprises 12,000 generation modules, the module cost per watt of power generated is approximately US$1.3. If the modules account for 75% of the total system cost, the entire system cost per kilowatt of power generated is US$1,733. If the cost breakdown remains the same, but the number of generation modules increases to 300,000 (which would be equivalent to 25 100-kW thermoelectric generation systems or a total generation of 2.5 MW), the module cost and total system cost would decrease to US$0.8/W and US$1,100/kW, respectively. If the selling price of a system were twice its production cost, the system would be sold at US$2,200 per kilowatt of power generated. According to the current investment and development of the green power industry, the estimated payback period and rate of return would still be reasonable. This estimation was made using the low generation efficiency of existing thermoelectric generation technology. Increasing the generation efficiency through technological development can further enhance the competitive advantage of thermoelectric generation. Therefore, in the development and industrialization of thermoelectric generation technology, reducing the cost of generation modules should be prioritized; the fastest method for achieving this goal would be to identify niche markets, increase user demand, and promote large-scale production.

### Case: evaluation of a 120-kW thermoelectric generation system

This case involved evaluating a waste processing plant in central Taiwan. The plant incinerates a large amount of waste, and the high-temperature exhaust generated as a consequence passes through a large heat exchanger before waste heat is recovered through heat transfer oil. The temperature of the recovered heat is approximately 230°C, and the flow rate of heat transfer oil is 300 m^3^/h. The heat transfer oil has a density of 729–737 kg/m^3^ and specific heat of 0.621–0.630 kcal/kg °C (210°C–230°C). Accordingly, when the difference in temperature before and after heat recovery is 11°C (230°C–219°C), the acquired thermal power is approximately 1.75 MW; when the temperature difference is 20°C (230°C–210°C), the acquired thermal power is 3.18 MW. Because a temperature difference of 20°C was acceptable to the plant owner, this condition was adopted for subsequent evaluation.

Because the heat source temperature is below 250°C, low-temperature BiTe TEG modules could be used. The heat exchanger was made of 6061 aluminum alloy. A measurement probe was used to examine the capacity of a thermoelectric generation system to generate power from the heat transfer oil and calculate the expected generation and heat exchange rate. The results revealed that the expected maximum generation of the system was 120 kW. The system was divided into five subsystems connected in parallel to form a power supply grid for the waste processing plant.

Table 1 presents the cost analysis of the case system. The table displays (1) system hardware costs (accounting for manufacturers’ profit), (2) machine maintenance costs, (3) the annual profit from power generation, and (4) the expected payback period. The annual profit from power generation was calculated using the mean industrial electricity price in Taiwan (US$0.08/kWh). Because the Taiwanese government provides a subsidy for installing waste heat recycling facilities, the maximum subsidy (one-third of the total facility cost, with an upper limit of US$166,667) was considered in the profit calculation. The results revealed that the expected payback period was approximately 3.7 and 5.43 years with and without the government subsidy, respectively, indicating that the 120-kW system, smaller than other commercial power generators, has a favorable cost recovery rate. Expanding the scale of the 120-kW system would further enhance its competitive advantages in terms of financial benefit.

**Table 1:** Estimated cost and payback period of the 120-kW thermoelectric generation system^87^

Nameplate capacity (kW)		120	Note
Operating hours (h)		8,000	
System establishment cost	A. Equipment price (US$)	400,000	–
	B. Expected subsidy (US$)	134,000	Government subsidy covers one-third of the implementation costs
	C. Installation cost (US$)	16,700	–
	D. Cost without subsidy (US$)	416,700	A + C
	E. Cost with subsidy (US$)	282,700	A + C-B
Maintenance cost	F. System maintenance (US$/y)	3,400	Mainly electrical control components
Energy conservation	Annual generation (kW)	960,000	–
	Electricity price (US$/kW)	0.08	–
	G. Electricity cost saved per year (US$)	80,000	–
	H. Net savings after deducting system maintenance cost (US$)	76,600	G-F
Payback period	Cost without subsidy divided by cost saved (year)	5.44	D/H
	Cost with subsidy divided by cost saved (year)	3.70	E/H

## Commercialization scale

This section details the construction of a ladder framework for commercializing and internationalization of a TEG-based low-temperature waste heat recovery facility. According to the commercialization and internationalization results reported by Symeonidou et al.,^88^ a product-based commercialization strategy will exhibit a low propensity for internationalization, but intellectual property (IP)-based commercialization can have a high intensity of internationalization. This study assumed that the TEG-based low-temperature WHRS had been developed and deployed worldwide. The IP-based strategy is employed by companies that provide related products. The information on 28 companies is summarized in Table 2. Among the 28 companies, there are some companies whose contribution to the waste heat recovery facilities is worth noting.

**Table 2:** Notable achievements and milestones of the companies working on WHRS

Name	Notable achievement	Milestone
ABB^89^	They provide WHRSs for large vessels that employ main propulsion machinery and have a more than 20 MW mechanical output, enabling more than the standard 50% fuel efficiency.	• Founded in 1988 • Initial Public Offering (IPO) at London and Frankfurt Stock Exchange in 2005
Alphabet Energy^90^	They partnered with Berkeley Lab to create a cost-effective thermoelectric WHRS. Using nanotechnology licensed by Berkeley Lab, they developed advanced thermoelectric materials based on silicon nanowires with a conversion efficiency of 10% or more and the ability to operate at temperatures as high as 800°C.	• First industrial-scale TEG, the E1 in 2014 • In 2017, the company received a US$2,000,000 grant from the California Energy Commission. • Closing down in 2019
European Thermodynamics^91^	They worked with companies across the globe, large and small, to deliver state-of-the-art thermal management solutions with TEGs.	• Founded in 2001 • At 2022, they announced the collaboration with Toyota.
Faurecia^92^	Faurecia has more than 10 years of experience with WHRS. They provide compact EHRS that weighs less than 3 kg and can reduce fuel consumption by up to 7%, thus improving fuel economy and reducing CO2 emissions.	• Founded in 1998 • In 2016, they equip the all-new Hyundai IONIQ Hybrid with WHRSs.
Ferrotec^93^	This company is a leading brand in providing materials and machinable ceramics for manufacturing TEGs.	• Founded in 1981 • In 2022, they prepared for Shenzhen IPO.
GreenTEG^94^	They provide energy-efficient thermoelectric elements based on the thermocouple technology developed by the Swiss Federal Institute of Zurich.	• Founded in 2009 • They raised a fund of CHF 10 million at 2022.
Hi-Z Technology^95^	They provide thermoelectric materials, devices, and systems. The first large generator was integrated into a diesel truck. Afterward, they continued to provide TEGs for exhaust or waste heat recovery at levels ranging from watts to kilowatts. They have been developing a 1 kW thermoelectric generator for class eight diesel truck engines. They also designed an mW generator for radio power supply.	• Founded in 1988 • They developed thermoelectric generators under U.S. DoE and California Energy Commission funding since 1992.
II-VI Marlow^96^	They offer thermoelectric coolers and cooling modules, power generation technology, and thermoelectric systems. The newest product is a wireless sensors’ thermal energy harvesting system. The Defense Advanced Research Projects Agency has used II-VI Marlow thermoelectric materials and devices.	• Founded in 1973 and named Marlow • In 2014, they became a division of II-VI Incorporated and is now known as II-VI Marlow.
iMEC^97^	It is a research center that can design WHRS. Case studies include NuWay Tobacco exhaust heat recovery, Vitasoy and Nasoya gas dryer heat recovery, Dirats Laboratories, Weetabix compressor, Smith’s medical hospital, and steam replacement with hot water at Freudenberg-NOK.	• A non-profit organization founded in 1984 • In 2022, they had more than 5,500 expert scientists from 96 countries.
Kelk^98^	They provide TEG modules for various applications. Their microgeneration module is made using their proprietary high-performance material and high-density element mount technology. The maximum temperature of these three models ranges from 80°C to 280°C, the electric resistance varies from 1.15 to 7 Ω, and the thermal resistance ranges from 1.048°C to 29°C/W	• Founded in 1966 • Sales of Mini-TEC started in 2002 • The multi-purpose model supports mercury-free since 2023.
Laird^99^	They provide TEGs to harness the energy and convert it into an efficient power source. The power generators designed by them are well suited for inaccessible locations and spaces.	• Founded in 1991 • They provided thermoelectric cooling for project laser since 2021.
Mitsubishi Heavy Industry^100^	They provide WHRs for container vessels. The main features of their WHRS are its easy operation, easy installation, high reliability, compact design, and that it is economical and environmentally friendly. The max power output of the WHRS ranges from 800 to 3,500 kW. The waste heat temperature is approximately 400°C.	• Founded in 1884 • Mitsubishi Heavy completed the first unit of the latest version of WHRS for marine engines in 2010.
Novus Energy^101^	They provide a high-temperature 50-W TEG module. The max efficiency is 9%, and the waste heat temperature can reach 800°C. The specific power at the module level is 2.8 kW/kg.	• Founded in 2004 • They raised a fund of USD 38 million at 2010.
Otego^102^	A spin-off company of Karlsruhe Institute of Technology. They developed a roll-to-roll manufacturing process to decrease the cost of TEGs and provide low-cost WHRS.	• Founded in 1948 • Joined Dickson Group in 1988 • The name changed to Otego in 2020
Perpetua^103^	Their Power Puck energy harvesters are compatible with the wireless sensors and transmitters used by leading providers of wireless industrial instrumentation.	• Founded in 2005 • They raised a found of USD 3 million at 2014.
Phononic Devices^104^	This company announced that they had harnessed the thermoelectric power of phonons to revolutionize how the world cools and heats. Phononic’s ENERGY STAR–certified refrigerators deliver up to 40% energy savings versus compressor-based refrigerators, with unprecedented temperature stability and uniformity that fluctuates only ±0.5°C.	• Founded in 2008 • Chief product officer for semiconductor firm since 2022 • They raised a fund with an undisclosed amount in 2014.
RGS Development^105^	They invent, develop, and manufacture new and sustainable TEGs based on nanotechnology.	• Founded in 2006 • Acquisition by Dow Corning RGS in 2010.
RIF Corporation^106^	They provide thermo-generating batteries (TBs) and thermoelectric gas generators. TBs convert thermal energy into electricity and are a finished product used for manufacturing self-contained electrical supply sources.	• Russian company founded in 2013 • Since 2018, they have provided products to over 44 companies.
RMT Ltd.^107^	RMT provides miniature thermoelectric coolers for telecom, industrial, medical, and aerospace applications. More than 2000 different thermoelectric coolers are available for temperature-stable control of precision equipment.	• Founded in 1994 • All thermoelectric coolers are RoHS compliant and Telcordia GR-468 qualified in 2020
Sango^108^	Their fourth-generation heat collectors (debuted in 2018) are 60% smaller in size and 41% lighter in weight than traditional TEGs.	• Founded in 1928 • Changed its name to Sango in 2021
TE Technology^109^	They offer a complete line of thermoelectric cooling modules, temperature controllers, Peltier coolers, and power supplies.	• Founded in 1989
TEGPro^110^	They provide TEG modules with approximately 5%–8% efficiency.	• Founded in 2011
Tellurex^111^	They provide TEGs for small dc devices. The net output of approximately 0.75 W is adjustable from 3 to 14 V dc.	• Founded in 1986 • Closing down in 2018
Tenneco^112^	They provide heat exchangers, TEGs, and Rankine cycle systems. With Tenneco’s one-box design, the Rankine cycle system is a comprehensive system that enables direct waste heat recovery from exhaust gas.	• Founded in 1940 • Reaching to revenue of USD 18 billion in 2021 and most of them from automotive products
Thermo Electric Company^113^	It is a multinational company specializing in designing and manufacturing temperature measurement solutions.	• Founded in 1941 • Thermo-Electric USA certification in 2020
Valeo^114^	Its thermal systems provide thermal climate control, thermal powertrain, thermal compressor, thermal front end, and waste heat recovery solutions.	• Founded in 1923 • In 2016 and 2017, Valeo ranked as France’s leading patent filer
Yamaha^115^	They contribute know-how in procuring and manufacturing thermoelectric modules made of semiconductor materials. At the same time, the WHRS helps with design, vehicle concepts, and optimizing vehicle energy systems in developing prototype thermoelectric-generator modules.	• Founded in 1987 • In 2021, Yamaha began to offer samples of YGPTX024 TEG module for vehicle use
Yasunaga^116^	Yasunaga is a Japan-based company mainly developing and selling engine parts, machinery, and environmental equipment. WHRSs were studied by this company.	• Founded in 1923 • Mg-Si system thermoelectric conversion material announced by Yasunaga at 2013

ABB company provides WHRSs for large vessels that employ main propulsion machinery and have a more than 20 MW mechanical output, enabling more than the standard 50% fuel efficiency.^89^ 2014 Alphabet Energy introduced the world’s first industrial-scale TEG, the E1. The E1 converts exhaust heat from large industrial engines into electricity.^90^ Ferrotec is a leading brand providing materials and machinable ceramics for manufacturing TEGs.^93^ Kelk company provides TEG modules for various applications. Their TEG modules are high performance and have the world’s highest conversion efficiency and high output power density. Their modules enable power generation equipment to be reduced in size.^98^ TEGpro company provides TEG modules with approximately 5%–8% efficiency. They provide the only thermoelectric generators made in the United States with US-made thermoelectric modules that can couple and uncouple from high-temperature heat sources.^110^

### Patent analytics

**Figure 8:**
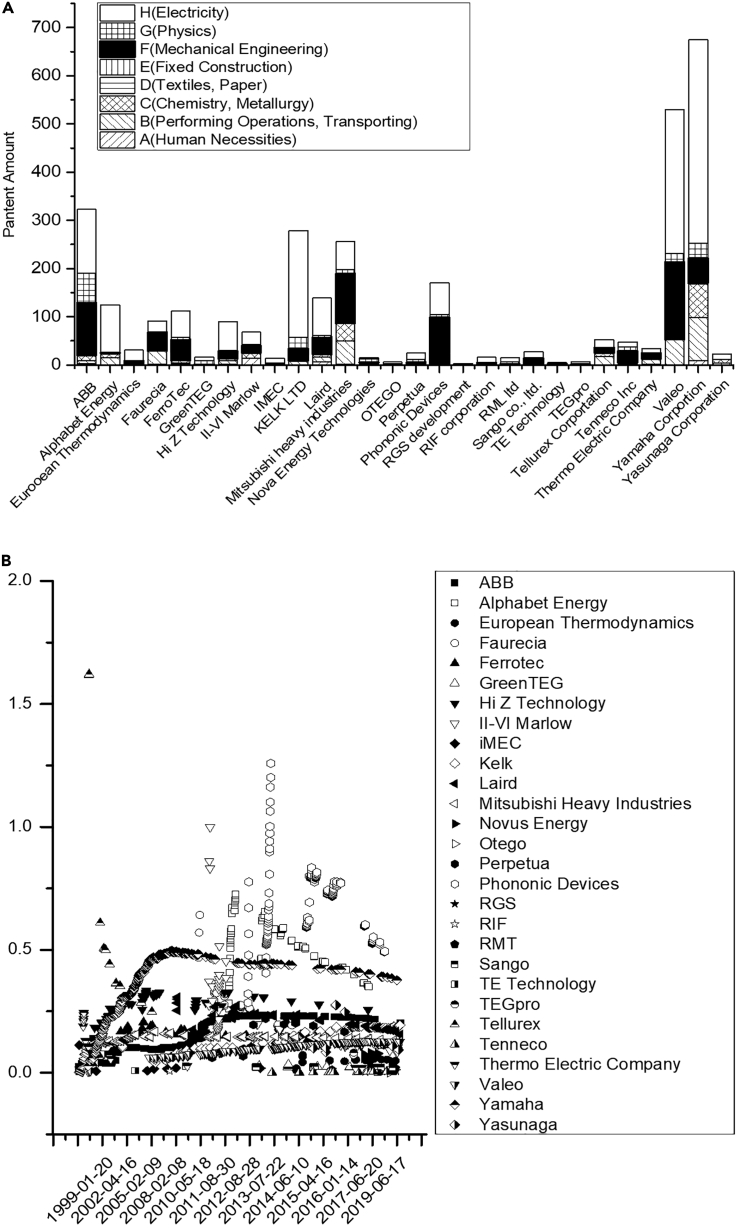
Patent analysis of thermoelectric systems (A) Number and IPC of TEG-based low-temperature WHRS-related patents. (B) PPI values of the 28 companies.

Figure 7C presents the preliminary analysis based on the International Patent Classification (IPC). Since they employed the IP-based commercialization strategy, this study assumed that the 28 companies owned influential patents. The research team investigated the companies’ patents on thermoelectric generators, the Seebeck effect, Peltier coolers, and waste heat. The results indicated that Yamaha Corporation has the highest of 454 patents. The second place is Valeo, with 387 patents. The third place is ABB, which owns about 281 patents. The quantity and types of patents each company owns are collected in Figure 8A. Among the companies’ TEG-based low-temperature WHRS-related patents, those related to electricity and mechanical engineering were the most common. Of the 2,365 patents, 1,247 were labeled “H” for electricity, and 591 were labeled “F” for mechanical engineering; these two types accounted for 77.8% of all patents. Additionally, 308 patents were labeled “B” for waste heat recovery methods, accounting for 9.6% of all patents. The remaining patents were mostly in the physics or chemistry categories, accounting for 12.6% of all patents. The preliminary IPC analysis indicated that TEG-based low-temperature WHRSs combine electricity and mechanical engineering technologies and are complemented by waste heat recovery techniques; these are the focus areas in developing TEG-based low-temperature WHRSs.

Most companies possess a considerable number of patents with “H” or “F” as the section code. The few exceptions included Alphabet Energy, which only applied for electricity-related patents. The company went bankrupt in 2019. However, because it was the only company in the analysis that went bankrupt, this did not constitute sufficient evidence to indicate that TEG-based WHRS suppliers must possess electricity and mechanical engineering patents to survive.

The IPC analysis based on the number and type of patents suggested that companies focusing on TEG-based WHRS development must file patents in the electricity and mechanical engineering fields to satisfy their research and development needs. This study developed a patent power analysis method to identify the main factors affecting product commercialization and international competitiveness. According to our previous study,^117^ the power of a patent can be analyzed using the Patent Power Index (PPI), which is defined as follows:

$$PPI=\frac{Count\;of\;citing+e^{\frac{153}{word\;amount\;of\;patent\;first\;claim}}}{Time\;after\;company\;found\;\left(per\;day\right)}$$

During a company’s developmental stage, PPI is utilized to track the value of patents quantitatively. The PPI is time variant. Therefore, PPI analysis can identify the time at which powerful patents appear. Such patents are then included in the patent landscape to determine how each company develops powerful patents to commercialize their products and compete in the international market. High product values under IP-based commercialization strategies are contingent heavily on the value of patent applications.^117^ Figure 8B presents the PPI values of the 28 companies.

Figure 8B also presents each company’s annual patent power variation. Examination of the background data of each company revealed that the companies offering WHRS components or modules possessed influential patents. However, these patents were concentrated in a single category, indicating that the companies owned relatively weaker patents in other categories. Tellurex, II-IV Marlow, and Phonic Devices possessed US1994207838A (thermoelectric module), GB201017159A (temperature control of electronic apparatus), and US2012643625P/WO2013US39945A (thermoelectric heat exchange system comprising cascaded cold-side heat sinks), respectively, which are the most fundamental patents for TEGs. The other companies, such as those providing whole systems, focused on patents with a specific value. For example, the patent with the highest PPI filed by Yamaha was JP1999199666A (thermoelectric element and its manufacturing method), by ABB was DE4340632A (switching control device), and by Valeo was FR201057877A WO2011EP63026A (method for manufacturing a thermoelectric device intended to generate an electric current in an automotive vehicle; Figure 8A). High PPI patents filed by other companies before 2020 related to these six patents were analyzed to construct the patent landscape.

The patents of the six companies mentioned in the previous paragraph constituted the basic framework of the patent landscape; with these fundamental patents, other relevant patents possessed by other companies were combined. This study focused particularly on the relevant patents owned by Alphabet Energy because its mean PPI value was higher than those of the other 27 companies. The high PPI patents owned by Alphabet Energy and included in this study were US13760977A (bulk nanohole structures for thermoelectric devices and methods of manufacture), US13299179A (arrays of long nanostructures in semiconductor materials and methods of manufacture), and US13749470A (modular thermoelectric units for heat recovery system and method of manufacture). High PPI patents related to these three patents filed by other companies were compared and analyzed to expand the patent landscape.

### Landscape of TEG-based WHRSs

**Figure 9:**
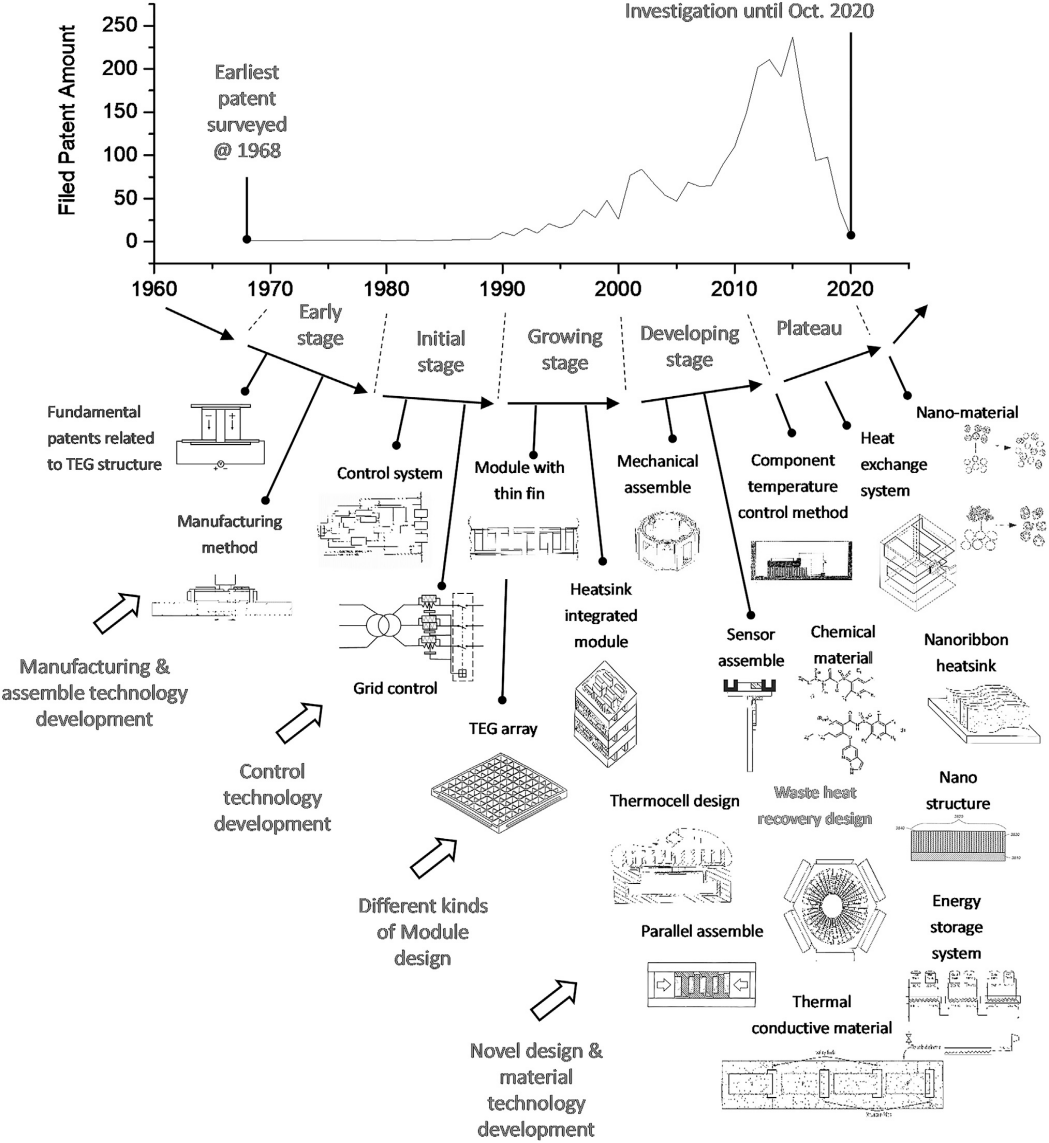
Landscape representing number of key patents with high PPI values

On the basis of the number of patents filed, the patent landscape can be used to evaluate a technology at different periods. The initial and early stages denote the periods when only a few patents are filed. The growing stage begins when the number of patents filed begins to increase. The developing stage denotes the period when the rate of patent applications increases. Finally, the plateau stage is reached when the number of patents reaches saturation. The patent landscape curve of the analyzed companies was plotted on a timeline, with the earliest patent surveyed dating to 1968, as shown in Figure 9.

Figure 9 shows the identified four crucial stages. The early stage occurred between 1970 and 1980, during which high PPI patents were mainly related to the manufacturing and assembly of thermoelectric chips for heat recovery. The initial stage occurred between 1980 and 1990, during which the commercialization of relevant products began. After the mechanical structures of TEG technology had been established, the initial development and integration of peripheral technologies, including control technology, thermal conduction modules, and grids, began. Electricity-related patents crucial to developing TEG-based WHRSs were all filed during this period. A rapid increase in the number of filed patents was observed between 1990 and 2000, during which the concept of modulation design emerged. The assembly of thermoelectric chips and heat transfer modules enhanced the quantity of heat recovery. During 2000–2010, many patents for sensors and novel heat transfer mechanisms were filed to increase heat recovery efficiency. After 2010, patents were mostly related to raw materials. Nanomaterials and microstructural heat exchangers were designed to increase heat recovery efficiency further. This study constructed a ladder framework based on the patent landscape.

### Ladder framework for commercialization

**Figure 10:**
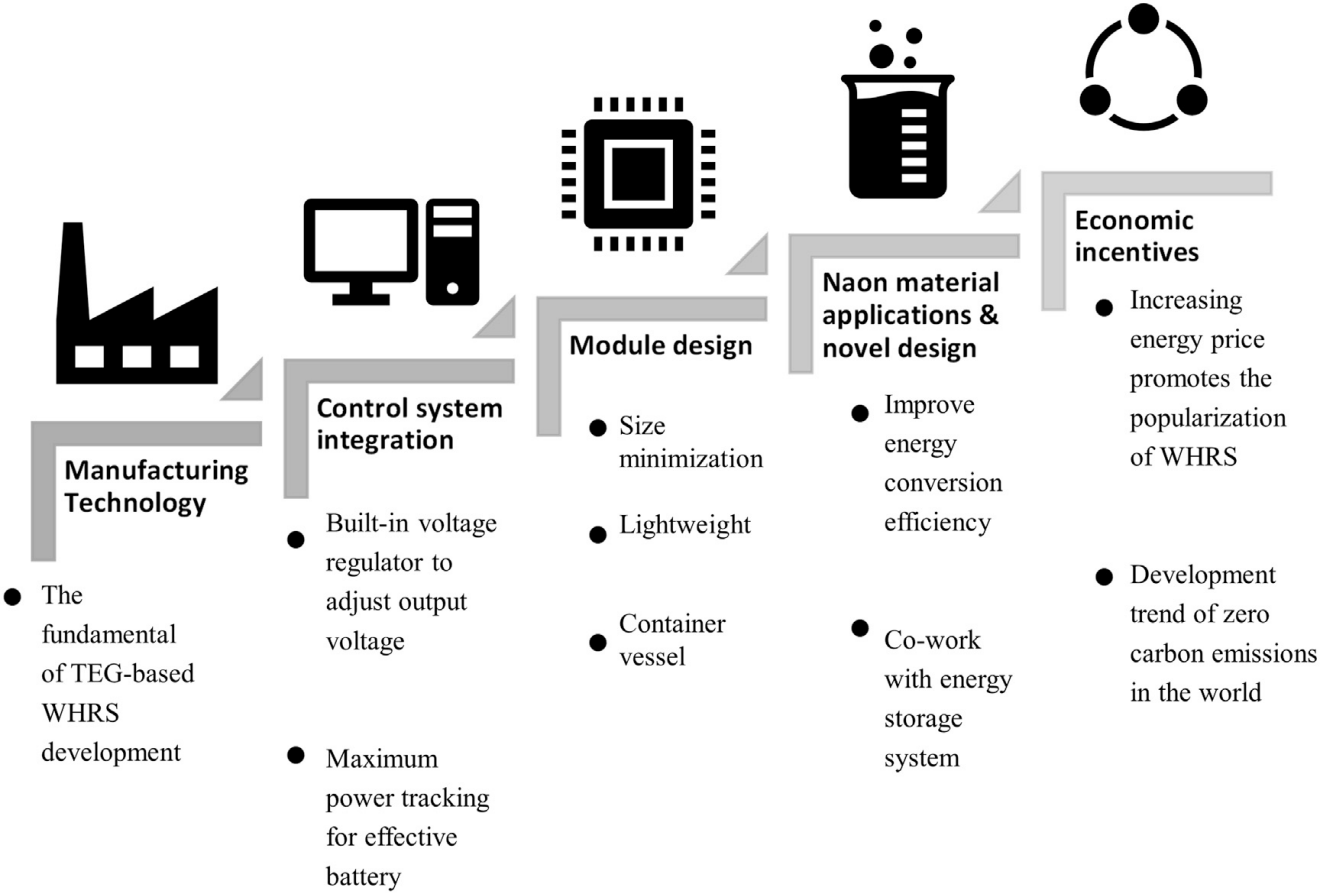
Ladder framework for TEG-based WHRSs

Figure 10 presents the ladder framework established on the basis of the development of thermoelectric generation technology. The TEG manufacturing technique is the foundation of the ladder framework. Mature manufacturing technology provides a solid foundation for developing waste heat recovery technology. The next hierarchical level in the ladder consists of control systems, particularly built-in voltage regulators and maximum power–tracking designs, which effectively convert waste heat to power output. Modular designs constitute the next hierarchical level. Such designs reduce equipment volume and weight and enable equipment to be packaged into container vessels. Accordingly, waste heat recycling can be achieved through simple modular assembly to reduce the overall cost. Nanomaterial applications and microstructural heat sinks were developed to further increase waste heat recovery efficiency. Energy prices and the global objective of zero carbon emissions are vital to promoting the commercialization and prevalence of relevant technology; these factors will determine the future development of WHRSs.

In addition to the conventional WHRS, some research institutions have developed flexible WHRS technology and plan to apply it to the field of wearable and portable flexible thermoelectric generators. Since their thermoelectric efficiency, durability, and cost are the bottlenecks for commercializing flexible WHRS, there are currently no commercial and marketable cases for wearable and portable flexible thermoelectric generators.^118^ In recent years, according to the latest research progress, the thermoelectric properties of flexible thermoelectric generators have been significantly affected by their materials and structures. In addition to thermoelectric properties, flexible thermoelectric generators’ mechanical properties and stability are important for their practical applications.^66^ In 2023, Zheng et al.^119^ stated that using a Bi_2_Te_3_ thin film design on the flexible thermoelectric generator, which can have high thermoelectric performance (the room temperature figure of merit ZT is about 1.2) and high flexibility (withstanding 2,000 bending tests at a bending radius of 8 mm). An exceptional output power density of 2.1 mW cm^−2^ is achieved by the flexible thermoelectric generators, which are made up of 40 pairs of thin films. This performance is demonstrated at a temperature gradient of 64 K, indicating that the device has the potential to be used for harvesting thermal energy from the environment or human bodies. The continual increase in energy prices worldwide and government subsidies to encourage zero carbon emissions are the two major financial incentives that can promote the large-scale commercialization of TEG-based WHRSs.

## Challenges

The development of thermoelectric generation for green power has received a great deal of attention, which is conducive to achieving fuel, resource, and environmental sustainability, as well as the target of net-zero emissions. Many international companies have deployed their patents on thermoelectric generation for commercialization, revealing the potential of this green power for marketization. However, a number of challenges are still encountered in the industrial application of thermoelectric generation. They are listed in the following section.

1. TEG patents were analyzed to construct a ladder framework for the commercialization and development of WHRSs. TEG manufacturing, modulation, materials, and energy conversion enhancement technologies have matured. The current challenges of large-scale commercialization of TEGs are not technological development but rather financial incentives associated with changes in international energy prices and subsidies to promote zero carbon emissions.
2. Zero carbon emissions can be achieved by providing subsidies to reduce the use of fossil fuels or increase the power generation efficiency of fossil fuel power stations. To achieve zero carbon emissions, the prevalence of TEG WHRSs must be increased, with data collected to verify such systems’ ability to increase overall power generation efficiency; governments must be encouraged to provide incentives for thermoelectric generation.
3. The technological structure of thermoelectric generation is simple. Because TEGs are heat exchangers capable of generating power, they are highly suitable for commercialization. Increasing thermoelectric conversion efficiency and decreasing thermoelectric material cost are the barriers to future large-scale commercial TEG development. In addition, ensuring the stability and durability of TEG materials is critical, particularly at high temperatures. Addressing these challenges would significantly broaden the potential applications of TEGs across multiple fields.

## Conclusion and outlooks

This study analyzed TEG patents filed by companies worldwide to identify fundamental technologies for achieving waste heat recovery through TEGs and explored the potential commercialization of relevant products. The IPC analysis revealed that most patents were associated with electricity and mechanical engineering technology. Mechanical engineering techniques are used to recover waste heat, and semiconductor and electrical technologies are used to convert thermal energy to electrical energy. The patent development of existing thermoelectric power generation technologies is saturated, so companies should focus on the practical implementation of relevant technologies to demonstrate that waste heat recovery through thermoelectric generation can offer good financial benefits. Short-term strategies for commercializing TEG-based WHRSs should focus on identifying niche markets and promoting government subsidies to increase the demand for TEG modules. This will reduce the cost of TEG modules and expand the market demand for TEG-based WHRSs, thereby creating a virtuous cycle for promoting relevant technologies.
